# Neurocognitive profiles in treatment-resistant bipolar I and bipolar II disorder depression

**DOI:** 10.1186/1471-244X-13-105

**Published:** 2013-04-04

**Authors:** Ute Kessler, Helle K Schoeyen, Ole A Andreassen, Geir E Eide, Åsa Hammar, Ulrik F Malt, Ketil J Oedegaard, Gunnar Morken, Kjetil Sundet, Arne E Vaaler

**Affiliations:** 1 Moodnet Research Group, Psychiatric Division, Haukeland University Hospital, Bergen, Norway; 2 Department of Clinical Medicine, Section of Psychiatry, University of Bergen, Bergen, Norway; 3 Moodnet Research Group, Psychiatric Division, Stavanger University Hospital, Stavanger, Norway; 4 Division of Mental Health and Addiction, Oslo University Hospital, Oslo, Norway; 5 Institute of Clinical Medicine, University of Oslo, Oslo, Norway; 6 Centre for Clinical Research, Haukeland University Hospital, Bergen, Norway; 7 Department of Global Public Health and Primary Care, University of Bergen, Bergen, Norway; 8 Department of Biological and Medical Psychology, University of Bergen, Bergen, Norway; 9 Department of Psychosomatic Medicine, Oslo University Hospital, Rikshospitalet, Oslo, Norway; 10 Department of Neuroscience, Faculty of Medicine, NTNU, Trondheim, Norway; 11 Division of Psychiatry, St. Olav’s University Hospital, Trondheim, Norway; 12 Department of Psychology, University of Oslo, Oslo, Norway

**Keywords:** Bipolar disorder depression, Cognitive functioning, MATRICS, IQ, Bipolar II disorder

## Abstract

**Background:**

The literature on the neuropsychological profiles in Bipolar disorder (BD) depression is sparse. The aims of the study were to assess the neurocognitive profiles in treatment-resistant, acutely admitted BD depression inpatients, to compare the neurocognitive functioning in patients with BD I and II, and to identify the demographic and clinical illness characteristics associated with cognitive functioning.

**Methods:**

Acutely admitted BD I (*n* = 19) and BD II (*n* = 32) inpatients who fulfilled the DSM-IV-TR criteria for a major depressive episode were tested with the MATRICS Consensus Cognitive Battery (MCCB), the Wechsler Abbreviated Scale of Intelligence, the National Adult Reading Test, and a battery of clinical measures.

**Results:**

Neurocognitive impairments were evident in the BD I and BD II depression inpatients within all MCCB domains. The numerical scores on all MCCB-measures were lower in the BD I group than in the BD II group, with a significant difference on one of the measures, category fluency. 68.4% of the BD I patients had clinically significant impairment (>1.5 SD below normal mean) in two or more domains compared to 37.5% of the BD II patients (*p* = 0.045). A significant reduction in IQ from the premorbid to the current level was seen in BD I but not BD II patients. Higher age was associated with greater neurocognitive deficits compared to age-adjusted published norms.

**Conclusions:**

A high proportion of patients with therapy-resistant BD I or II depression exhibited global neurocognitive impairments with clinically significant severity. The cognitive impairments were more common in BD I compared to BD II patients, particularly processing speed. These findings suggest that clinicians should be aware of the severe neurocognitive dysfunction in treatment-resistant bipolar depression, particularly in BD I.

**Trial registration:**

Trial registration number: NCT00664976

## Background

Bipolar disorder (BD) is associated with various cognitive impairments, of which deficits in verbal learning, attention, and executive functions are the most frequently reported [1-3]. The literature on the neuropsychological profiles in BD depression is sparse [4-9]. Studies on cognitive function have often not distinguished between BD and recurrent depressive subgroups, or else they have involved heterogeneous patient groups with BD in euthymic, mixed, or unclassified mood states. A meta-analysis of studies on cognitive function in euthymic, manic or mixed, and depressed BD patients [10] revealed cognitive impairments in all phases of the illness, across all neuropsychological domains, with a moderate worsening of a subset of deficits in acute states.

BD I and BD II patients present with heterogeneous clinical symptoms [11]. In contrast to the Diagnostic and Statistical Manual of Mental Disorders, Fourth Edition, Text Revision (DSM-IV-TR) [12], BD II does not exist as a specific diagnosis in the International Classification of Diseases, Tenth Revision (ICD-10) [13]. There are arguments for and against BD II as a distinct diagnostic entity [14]. Comparing the neuropsychological functioning between the BD I and BD II types of depression may provide indications of these putative different entities. Several studies have compared cognitive functioning in euthymic BD I and BD II patients [15-18], and deficits were found in both groups. Most studies have found more cognitive deficits in BD I patients, with the most prominent difference being in memory function. However, in a limited number of BD patients with current mood state euthymia or mild depression recruited from out-patient clinics or journal advertisements, Summers and colleagues found that patients with BD I performed better than those with BD II [19]. We are aware of only one study comparing the neuropsychological performance in depressed unipolar, BD I and BD II patients [20]. Xu et al. found a similar pattern of cognitive impairment in the three groups, with cognitive dysfunction in processing speed, memory, verbal fluency and executive functioning, but not attention. BD I patients were more impaired than BD II and unipolar depressed patients in verbal fluency and executive function. There are indications of a possible neurodegenerative process in BD [21]. More extensive cognitive impairment may be associated with a more severe course of illness, including a greater number of episodes, history of psychotic symptoms, and longer duration of illness [22-24]. A previous study found a correlation between a reduction in IQ and structural changes in BD [25].

Treatment resistant BD depression patients constitute a significant proportion of inpatient samples. These patients represent a clinical challenge. Further research on cognitive functioning may have impact on factors affecting acute treatment and follow up.

### Aims of the study

The main aims of the present study were to assess the neurocognitive profiles in treatment-resistant, acutely admitted BD depression inpatients, to compare the neurocognitive functioning in patients with BD I and II, and to identify the demographic and clinical illness characteristics associated with cognitive functioning.

## Methods

### Study design

The data were collected in the Norwegian randomized controlled trial of electroconvulsive therapy in acutely admitted, treatment-resistant BD inpatients. The protocol for this trial has been published previously [26]. The neuropsychological functioning at baseline was assessed after admittance to hospital but before the start of treatment.

### Subjects

The study participants comprised 51 patients who met DSM-IV-TR [12] criteria for BD I (*n* = 19) or BD II (*n* = 32) disorder. The diagnosis was made primarily on the basis of a clinical interview supported by information from significant others and hospital records, and subsequently verified by the Mini-International Neuropsychiatric Interview (MINI; specifically the MINI-Plus) [27] or the Structured Clinical Interview for DSM-IV Axis I Disorders (SCID-I) [28]. The assessing psychiatrists had participated in a structured training program for SCID-I or MINI-Plus.

The patients fulfilled the DSM-IV-TR criteria of a depressive episode [12]. The severity of depressive symptoms was assessed using the Montgomery and Åsberg Depression Rating Scale (MADRS) [29], with a cut-off score of ≥25 for participation in the study. All patients were treatment resistant in terms of a nonresponse to at least two lifetime trials with mood stabilizers with proven efficacy in BD depression (lithium, lamotrigine, quetiapine, and olanzapine) and/or antidepressants. A trial was defined as a minimum of 6 weeks on an adequate or tolerated dose as reported by the patient, or for a shorter period when treatment was terminated prior to 6 weeks due to side effects. Nonresponse was defined as a reduction in MADRS scores of <50% or still fulfilling DSM-IV-TR criteria of a depressive episode. Patients had to be sufficiently fluent in Norwegian to ensure valid responses to psychometric testing (i.e., Norwegian must be their primary language, or they must have received their compulsory schooling in Norwegian).

The criteria for exclusion were treatment with electroconvulsive therapy (ECT) within the previous 6 months or having been diagnosed with conditions that may affect neuropsychological assessment, such as Parkinson’s disease, multiple sclerosis, stroke, ongoing alcohol or substance abuse, or other criteria as defined in the protocol [26].

### Assessment of symptoms

Symptom intensity was rated using the MADRS [29], the Positive and Negative Syndrome Scale for Schizophrenia, positive subscale (PANSS pos) [30], and the Global Assessment of Functioning–Split version, symptom subscale (GAF-S) [31]. Symptoms were assessed by trained clinicians (psychiatrists, psychologists, and psychiatric nurses).

### Definition of illness characteristics

The patients were interviewed with the Norwegian adaptation of the Stanley Foundation Bipolar Collaboration Network Entry Questionnaire (NEQ) used by the Bipolar Collaboration Network [32-35]. The NEQ has 48 items and covers a wide range of demographic and clinical factors describing the course of illness. Substance abuse was defined as fulfilling the DSM-IV-TR criteria for lifetime abuse of alcohol, psychotropic medication, or illicit substances. Psychosis was defined as lifetime admission to hospital with a psychotic illness, as verified by the MINI-Plus or the SCID-I. Length of education was quantified as the duration of completed education in years. Previous serious suicide attempts were defined as attempts that required medical attention, an emergency room visit, or hospitalization [36].

### Neuropsychological assessment

Neurocognitive assessment was carried out by clinical psychologists or test assistants with training in standardized neuropsychological testing. Current IQ was assessed using the Wechsler Abbreviated Scale of Intelligence (WASI) [37]. The National Adult Reading Test (NART) [38] is designed to estimate the premorbid intelligence in adults. Reading skills are significantly correlated with Wechsler-based IQ scores and is relatively unaffected by most nonaphasic brain disorders [39]. In the present study, the premorbid IQ was estimated with a Norwegian research version of the NART [40].

The Measurement and Treatment Research to Improve Cognition in Schizophrenia (MATRICS) Consensus Cognitive Battery (MCCB) [41] is designed for use in clinical trials assessing cognitive function in schizophrenia and related psychiatric disorders [42]. Although there is no established standard battery specifically designed to assess cognition in BD, recent papers [43,44] support the use of MCCB in BD research. The neuropsychological profile (consisting of the six neurocognitive domains, I–VI, listed below) was assessed using the following nine tests from the Norwegian version [45] of the MCCB [41]:

I. **Speed of processing:**

1. Brief Assessment of Cognition in Schizophrenia (BACS): Symbol Coding (total number correct).

2. Category Fluency: Animal Naming (total number of animals named in 60 seconds).

3. Trail-Making Test: part A (TMT-A): (time to completion).

II. **Attention/Vigilance:**

4. Continuous Performance Test-Identical Pairs (CPT-IP): (mean d’ value across two-, three-, and four-digit conditions, where d’ is an index of signal–noise discrimination).

III. **Working memory:**

5. Wechsler Memory Scale–third edition (WMS-III): Spatial Span (sum of raw scores on the forward and backward conditions).

6. Letter-Number Span: (total number correct).

IV. **Verbal learning:**

7. Hopkins Verbal Learning Test–Revised (HVLT-R): (total number of words recalled correctly over three learning trials).

V. **Visual learning:**

8. Brief Visuospatial Memory Test–Revised (BVMT-R): (total recall score over three learning trials).

VI. **Reasoning and problem solving:**

9. Neuropsychological Assessment Battery (NAB): mazes (total raw score).

Raw scores from each of the nine administered MCCB tests were converted into standardized T-scores with a mean of 50 and standard deviation of 10, based on age- and gender-corrected norms from the MCCB manual [42]. The T-scores for the six assessed domains were used to compute a mean neurocognitive composite score. Nine patients had missing scores in one, and one patient in two of the tested domains. For these ten subjects, the mean composite score was computed based on the remaining domain T-scores.

### Statistical analysis

Characteristics for the BD I and BD II patients were compared using *t*-tests and analysis of variance for continuous, and chi-square test for categorical variables. Statistical comparisons between BD subtypes for non-normally distributed variables were performed with a Mann-Whitney Test. Correlation and multiple linear regression analyses were performed between neuropsychological measures and demographic variables (gender, age, education, and premorbid IQ), course of illness (BD subtype, duration of illness, number of hospitalizations due to depressive episodes, number of psychotic episodes, history of psychosis, comorbid substance abuse, and comorbid anxiety), and current symptoms (MADRS, PANSS pos, GAF-S). Due to the small number of patients relative to the large number of independent variables, analyses were conducted unadjusted, and adjusted for age and education only*.* The level of statistical significance was set at *p* < 0.05. All statistical analyses were performed using the Statistical Package for the Social Sciences (version 18.0, SPSS, Chicago, IL, USA).

### Ethics

The study was approved by The Regional Committee for Research Ethics, Central Norway, and The Norwegian Data Inspectorate. The study is registered in the online clinical database ClinicalTrials.gov (identifier http://NCT00664976). All subjects provided informed written consent to participate prior to their inclusion in the study.

## Results

### Demographic characteristics

The demographic characteristics of the BD I and BD II patients are listed in Table 1. The BD I group had a shorter duration of education and comprised more patients with a history of psychosis compared to the BD II group. The estimated premorbid IQ did not differ between the groups, both of which performed in the “above normal” range. The scores on the rating scales used to assess symptom severity did not differ between the groups.

**Table 1:** Clinical characteristics of bipolar disorder (BD) inpatients with therapy-resistant depression

**Variable**		**Total sample**	**BD I**	**BD II**	***p***
			***n*** **= 19**	***n*** **= 32**	
Demographics	Gender (male), *n* (%)	24 (47.1)	7 (36.8)	17 (53.1)	0.20^1)^
	Age (years)	45.7 (10.5)	49.2 (9.2)	43.6 (10.8)	0.06^2)^
	Education (years)	13.8 (3.2)	12.6 (3.2)	14.5 (3.0)	**0.04**^2)^
	Premorbid IQ	112.9 (3.7)	112.0 (3.9)^a^	113.5 (3.4)^b^	0.18^2)^
Course of illness	Duration of illness (years)	30.6 (11.4)	33.4 (9.4)^a^	29.1 (12.2)	0.19^2)^
	Hospitalizations due to depressive episodes	4.3 (3.9)	4.7 (4.8)^c^	4.1 (3.5)^d^	0.82^3)^
	History of psychotic symptoms, *n* (%)	24 (48.0)	16 (84.2)	8 (25.8)^e^	**<0.01**^1)^
	Number of psychotic episodes	1.8 (3.0)	3.6 (3.6)^c^	0.8 (2.1)^f^	**<0.01**^3)^
Lifetime DSM-IV-TR diagnosis	Substance abuse, *n* (%)	15 (29.4)	6 (31.6)	9 (28.1)	1.00^1)^
	Anxiety, *n* (%)	14 (29.2)	8 (47.1)^g^	6 (19.4)^e^	0.06^1)^
Symptom rating scales	MADRS	37.0 (6.6)	36.8 (6.6)	37.2 (6.6)	0.87^2)^
	PANSS pos	9.8 (3.4)	9.7 (3.6)^c^	9.8 (3.4)	0.76^3)^
	GAF-S	37.3 (10.9)	41.0 (9.8)^a^	35.3 (11.1)	0.10^3)^

Data are mean (SD) values unless otherwise specified for total group and groups with BD I or BD II subtype, accompanied by *p* values for statistical comparisons between subtypes: ^1)^chi-square test; ^2)^independent-samples *t*-test; ^3)^Mann-Whitney Test. Data presented in bold, *p* < 0.05.

*SD*, Standard deviation; *MADRS*, Montgomery and Åsberg depression rating scale; *PANSS pos*, Positive and negative syndrome scale for schizophrenia, positive subscale; *GAF-S*, Global assessment of functioning–split version, symptom subscale; *DSM-IV-TR*, Diagnostic and statistical manual of mental disorders, fourth edition, text revision.

^a^*n* = 18, ^b^*n* = 28, ^c^*n* = 15, ^d^*n* = 30, ^e^*n* = 31, ^f^*n* = 29, ^g^*n* = 17.

Two patients reported an alcohol consumption of more than 3 units/day (where 1 unit = approximately one glass of wine or one bottle of beer), and four reported sporadic use of amphetamine, heroin, or cannabis during the 6 months preceding inclusion. Previous serious suicide attempts were reported by 56%, with no significant difference between the groups.

At admittance, 64% of the patients used second-generation antipsychotics, 50% used antiepileptics, 74% used antidepressants, 30% used lithium, 48% used benzodiazepines, 86% used a psychotropic combination, and 6% used no psychotropic medication; these proportions did not differ significantly between the groups.

### Neurocognitive profile

Age- and gender-corrected T-scores for the nine cognitive MCCB tasks, the six domain scores and mean composite score, current IQ assessed using the WASI, and decrease in IQ from the estimated premorbid IQ assessed using the NART, are listed in Table 2. The numerical scores on all measures were lower in the BD I group than in the BD II group, but the difference was significant on only one of the measures, category fluency. The effect sizes for these differences are classified as small to medium [46]. The performance on the WASI was significantly worse for the BD I patients than for the BD II patients. This indicates a decline in IQ in the BD I patients from the premorbid to the current level. The IQ declines in the BD I and BD II groups were 18.0 and 6.1, respectively (*p* < 0.01). The effect sizes for the differences in current IQ and IQ decline are both classified as large.

The MCCB profiles for the six cognitive domains in the BD I and BD II groups indicate neurocognitive functioning at a level between 1 and 1.5 SD below normal means across domains. The performances in both groups were significantly below the normal means for all domains. Patients obtaining scores lower than 1.5 SD below the normal mean (i.e., T ≤ 35) are classified as clinically impaired. The percentages of patients with impairment in the different domains are listed in Table 3. The percentage of impaired patients was higher in the BD I group than in the BD II group, with significant differences in speed of processing, current IQ, and IQ decline. Clinically impairment was more common among BD I patients: the percentage of BD I and BD II patients with neurocognitive impairments in two or more domains were 68.4% and 37.5%, respectively (chi-square test: *p* = 0.045).

### Influence of demographic and illness characteristics on cognitive functioning

**Figure 1:**
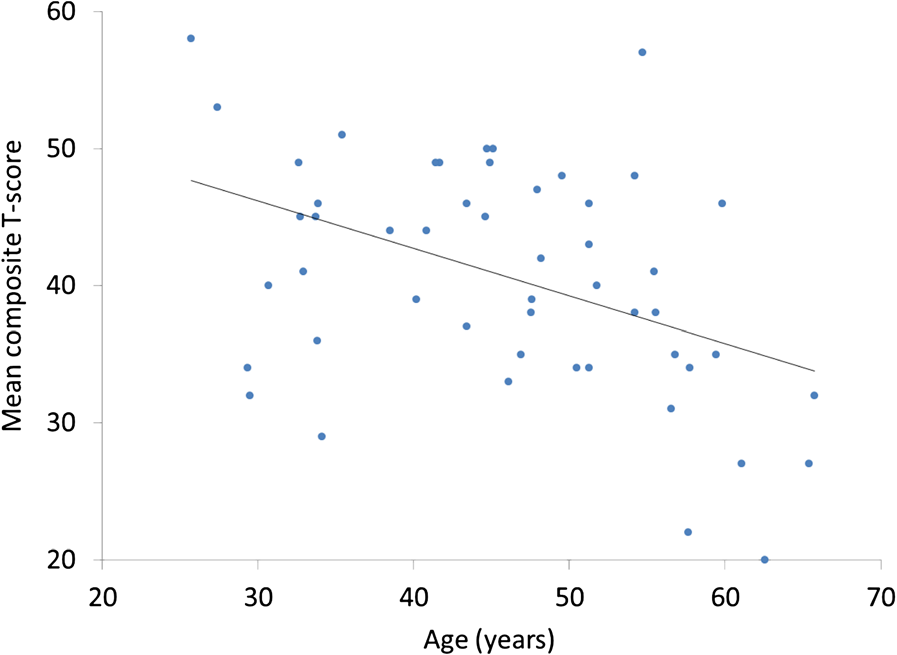
**Relationship between mean composite T-score and age for 51 inpatients with treatment-resistant depression in bipolar disorder.** Note: The mean composite score is based on six domain scores from nine cognitive tasks from the MATRICS Consensus Cognitive Battery.

The associations between demographic and illness characteristics and cognitive measures are listed in Table 4. We found a significant correlation between age and test performance. The expected cognitive decline associated with normal aging was accounted for by using age-adjusted T-scores. Higher age was associated with an increased difference from the normal mean (T = 50). The relationship between the mean composite score and age is shown in Figure 1. Age and education strongly influenced test performance, and these two variables were controlled for in a multiple linear regression analysis. Neither course of illness (diagnostic subtype, number of hospitalizations due to depressive episodes, number of psychotic episodes, history of psychosis, comorbid substance abuse, and comorbid anxiety disorder) nor level of symptoms (MADRS, PANSS pos, and GAF-S scores) were associated with overall cognitive function (mean composite score) or the six cognitive domains when controlled for age and education (details not shown). Diagnostic subtype BD I (*b* = 9.2, *p* = 0.02) and history of psychosis (*b* = 9.1, *p* = 0.01) were associated with a greater IQ decline when controlled for age and education, whereas longer duration of illness was negatively associated to IQ decline (*b* = -0.9, *p* = 0.002) when controlled for age and education.

**Table 2:** Test performance (T-scores) for the total sample, BD I and BD II subsamples in inpatients with therapy-resistant BD depression on nine cognitive tasks from the MCCB, six domain scores and mean composite score, and IQ, and change in IQ from the estimated premorbid level

**Neurocognitive domain**	**Total sample**	**BD I**	**BD II**	**ANOVA**		
** Test**	***n*** **= 51**	***n*** **= 19**	***n*** **= 32**	***F***	***p***	***η***^**2**^
	**Mean (SD)**	**Mean (SD)**	**Mean (SD)**			
Speed of processing	36.5 (12.3)	32.3 (14.6)	39.0 (10.2)	3.69	0.06^1)^	0.07
BACS–Symbol Coding	37.0 (11.7)	35.0 (13.6)	38.2 (10.5)		0.67^2)^	
Fluency	44.7 (9.5)	40.7 (10.5)	47.1 (8.1)	5.91	**0.02**^1)^	0.11
TMT-A	37.7 (13.1)	33.7 (13.5)	40.0 (12.6)	2.77	0.10^1)^	0.05
Attention/Vigilance	40.7 (11.4)^a^	38.5 (11.8)^b^	41.8 (11.3)^c^	0.78	0.38^1)^	0.02
CPT						
Working memory	43.5 (11.2)	41.2 (11.1)	45.0 (11.3)	1.37	0.25^1)^	0.03
Spatial Span	45.9 (10.6)^d^	44.2 (10.8)	47.0 (10.5)^e^	0.80	0.38^1)^	0.02
Letter-Number Span	43.5 (10.7)	41.2 (10.7)	44.9 (10.7)	1.45	0.24^1)^	0.03
Verbal learning	38.4 (8.1)	36.5 (7.6)	39.5 (8.2)	1.73	0.19^1)^	0.03
HVLT-R						
Visual learning	43.0 (11.7)^f^	40.7 (13.7)^g^	44.3 (10.4)^h^	1.04	0.31^1)^	0.02
BVMT-R						
Reasoning	42.3 (9.3)^d^	41.9 (7.8)^g^	42.4 (10.1)		0.81^2)^	
Mazes						
Mean composite score	40.6 (8.4)	38.4 (9.3)	41.9 (7.7)	2.19	0.14^1)^	0.04
IQ	102.3 (13.6)	93.9 (15.2)	107.2 (10.2)	14.05	**<0.01**^1)^	0.22
WASI						
IQ difference	10.8 (13.0)^i^	18.0 (13.7)^g^	6.1 (10.3)^j^	11.29	**<0.01**^1)^	0.20
NART-WASI						

Note: Scores on the MCCB measures have a mean of T = 50 and an SD of T = 10. IQ scores have a mean of 100 and an SD of 15. *p* values for statistical comparisons between BD subtypes: ^1)^ANOVA; ^2)^Mann-Whitney Test. Data presented in bold, *p* < 0.05.

*MCCB*, Measurement and treatment research to improve cognition in schizophrenia (MATRICS) consensus cognitive battery; *ANOVA*, Analysis of variance; *BACS*, Brief assessment of cognition in schizophrenia; *TMT-A*, Trail-making test, part A; *CPT*, Continuous performance test; *HVLT-R*, Hopkins verbal learning test–revised; *BVMT-R*, Brief visuospatial memory test–revised; *WASI*, Wechsler abbreviated scale of intelligence; *NART*, National adult reading test.

^a^*n* = 44, ^b^*n* = 14, ^c^*n* = 30, ^d^*n* = 50, ^e^*n* = 31, ^f^*n* = 49, ^g^*n* = 18, ^h^*n* = 31, ^i^*n* = 46, ^j^*n* = 28 due to missing data.

## Discussion

The present findings indicate that patients with therapy-resistant BD depression exhibit reduced performance in all of the cognitive domains assessed by the MATRICS battery. Almost half of the patients were impaired in two or more domains, indicating that cognitive deficits are relatively common and non-specific in BD depression. We found that speed of processing and verbal learning were the most frequently and severely affected domains. Speed of processing was also found to be the most severely affected domain in a study using the MCCB in euthymic and symptomatic BD I patients [44]. There is only one test in the MCCB assessing executive functioning; mazes. That may explain why both in the current study and in the study by Burdick et al. executive functioning seemed to be less impaired than previously reported [47].

In the present study, the percentage of patients with clinically significant cognitive impairment varied across domains and measures, but it was higher than previously reported for stable outpatients [16]. Clinically significant cognitive impairment, defined as ≥1.5 SD below the control group mean, was reported for 24% of BD I and 13% of BD II outpatients [16], versus 42% and 25%, respectively, in the present study. This difference may be attributable to the specific group of treatment-resistant, acutely admitted depressed inpatients.

**Table 3:** Percentage of patients with cognitive impairment^* ^**for the total sample of 51 inpatients with therapy-resistant BD depression, BD I, and BD II on nine cognitive tasks from the MCCB, six domain scores and mean composite score, and IQ and change in IQ from the estimated premorbid level**

**Neurocognitive domain**	**Total sample**	**BD I**	**BD II**	**BD I vs. BD II**	
** Test**	***n*** **= 51**	***n*** **= 19**	***n*** **= 32**	***χ***^**2**^	***p***
Speed of processing	43.1	63.2	31.2	4.9	**0.041**
BACS–Symbol Coding	49.0	52.6	46.9	0.2	0.776
Fluency	17.6	31.6	9.4	4.0	0.062
TMT-A	45.1	63.2	34.4	4.0	0.080
Attention/Vigilance	36.4^a^	42.9	33.3	0.4	0.738
CPT					
Working memory	21.6	21.1	21.9	0.0	1.000
Spatial Span	18.0^b^	26.3	12.9	1.4	0.273
Letter-Number Span	25.5	31.6	21.9	0.6	0.515
Verbal learning	39.2	42.1	37.5	0.1	0.774
HVLT-R					
Visual learning	24.5^c^	38.9	16.1	3.2	0.094
BVMT-R					
Reasoning	30.0^b^	22.2	34.4	0.8	0.523
Mazes					
Mean composite score	31.4	42.1	25.0	1.6	0.228
IQ	5.9	15.8	0.0	5.4	**0.047**
WASI					
IQ decline (NART-WASI) ≥1.5 SD	15.2^d^	33.3	3.6	7.5	**0.010**

^*^Defined as at least 1.5 SD below the normal mean.

^a^*n* = 44, ^b^*n* = 50, ^c^*n* = 49, ^d^*n* = 46 due to missing data.

With scores on cognitive measures 1-1.5 SD below normal mean the patients in the current sample experienced somewhat greater cognitive deficits than described in euthymic [47,48], or depressive state [8]. Although the patients in the current study tended to perform slightly weaker than reported from other patient samples, we did not find substantial differences in the cognitive profile. This is in line with the findings from Kurtz and Gerraty [10], describing reduced cognitive function in all phases of BD, across all neuropsychological domains, and a moderate deterioration of some measures in acute state.

Previous findings from mainly euthymic BD patient populations indicate a significant difference in cognitive performance between BD I and BD II patients, with BD I patients being more affected [15,16]. The present study yielded a similar trend but it was not statistically significant—this may have been due to the sample size.

The BD patients did not exhibit impairment in premorbid IQ, which is consistent with previous findings [49]. We found a significant difference in IQ decline between BD I and BD II patients, with 33% and 4%, respectively, showing a decline in IQ of at least 1.5 SD (22.5 points). The findings of the present study suggest that there are neurobiological differences between BD I and BD II. We are not aware of any other reports of a similar IQ decline restricted mainly to the BD I population. However, one study involving euthymic and depressed outpatients [19] found an IQ decline in 2 out of 11 BD II patients and none of the BD I patients. These conflicting results might be due to differences in patient recruiting; whereas the present study assessed treatment resistant BD patients in a major depressive episode, Summers and colleagues evaluated euthymic and mildly depressed patients recruited from out-patient clinics and through journal advertisements.

A total of 48% of the patients in the present study had experienced prior psychotic episodes (84% of BD I patients and 26% of BD II patients). The impact of the prior psychotic symptoms on cognitive measures in BD has been investigated in several studies, and a meta-analysis [24] concluded that a prior history of psychotic symptoms is associated with increased impairments in several cognitive domains. We found an association between the number of previous psychotic episodes and IQ decline from premorbid to current levels. However, we did not find an association between the number of psychotic episodes and MCCB scores. We found a negative correlation between illness duration and IQ decline when we controlled for age and education. This might be due to larger IQ decline for patients with an older age at onset.

**Table 4:** Correlations between demographic and illness characteristics and neurocognitive performance for the total sample of 51 inpatients with therapy-resistant BD depression

**Neurocognitive domain**	**Demographic characteristics**				**Course of illness**							**Current symptoms**		
	**Gender (male)**	**Age**	**Education**	**Premorbid IQ**	**BD I**	**Duration of illness**	**Number of hospitalizations due to depresssive episodes**	**History of psychotic symptoms**	**Number of psychotic episodes**	**Comorbid substance abuse**	**Comorbid anxiety**	**MADRS**	**PANSS pos**	**GAF-S**
** Test**				**(NART)**										
Speed of Processing	-0.09	-0.21	0.25	0.20	-0.25	-0.12	-0.02	-0.14	-0.10	0.09	0.02	0.05	-0.01	-0.05
BACS–Symbol Coding	-0.19	-0.29^*^	0.28	0.27	-0.06	-0.27	-0.14	-0.04	0.01	0.08	0.17	-0.06	-0.15	0.06
Fluency	-0.09	-0.11	0.20	0.06	-0.35^*^	0.10	0.22	-0.20	-0.24	0.01	0.05	-0.01	-0.05	0.17
TMT-A	-0.02	-0.19	0.23	0.14	-0.22	-0.15	-0.11	-0.09	-0.11	-0.01	-0.09	0.11	0.09	-0.22
Attention/Vigilance	-0.20	-0.46^**^	0.14	0.00	-0.11	-0.43^**^	-0.29	-0.08	-0.10	0.23	0.20	-0.19	-0.06	-0.10
CPT														
Working memory	-0.17	-0.43^**^	0.36^**^	0.16	-0.13	-0.27	-0.01	-0.07	-0.07	0.04	0.19	0.01	-0.20	0.05
Spatial Span	-0.10	-0.42^**^	0.27	0.13	-0.12	-0.21	0.09	0.07	0.08	0.12	0.32	0.10	-0.20	0.00
Letter-Number Span	-0.17	-0.38^**^	0.37^**^	0.16	-0.11	-0.31^*^	-0.10	-0.20	-0.20	-0.05	0.05	-0.05	-0.15	0.06
Verbal learning	-0.04	-0.37^**^	0.32^*^	0.36^*^	-0.14	-0.25	-0.05	-0.20	-0.14	0.05	0.01	0.00	-0.25	0.02
HVLT-R														
Visual learning	-0.09	-0.28	0.34	0.16	-0.21	-0.14	0.17	-0.17	-0.12	0.12	0.23	-0.04	-0.14	0.03
BVMT-R														
Reasoning	-0.21	-0.19	0.22	0.04	0.04	-0.06	-0.13	0.10	0.08	0.18	0.21	0.07	0.02	-0.04
Mazes														
Mean Composite score	-0.18	-0.40^**^	0.36^**^	0.22	-0.19	-0.26	-0.07	-0.13	-0.12	0.10	0.15	-0.02	-0.13	-0.04
IQ-decline	-0.21	0.17	-0.41^**^	-0.17	0.43^**^	-0.05	0.17	0.40^**^	0.41^*^	-0.17	-0.01	0.05	0.26	-0.17
NART-WASI														

Spearman’s rho is reported. ^*^p < 0.05; ^**^p < 0.01.

Studies of associations between depression severity and cognitive impairment in BD have produced contradictory results, with one study [50] finding that mildly depressed patients performed better on cognitive tasks than severely depressed patients, whereas studies involving outpatients [51] or mainly euthymic BD I patients [52] found no relationship between cognitive measures and depressive symptoms. The present findings indicate that cognitive impairments were correlated more strongly with age and education than with symptom severity in inpatients with treatment-resistant BD depression. The non-significant correlation between symptom severity and cognitive impairment in this study might be due to small variance, since all the participants were severely affected.

There is a cognitive decline in normal aging. This was accounted for by using age-adjusted T-scores. The present cross sectional findings of the cognitive deficits increasing with age are consistent with a longitudinal study documenting a greater cognitive decline over 3 years in older BD patients compared to healthy controls with the same age and education [53], whereas a study of 65 euthymic elderly outpatients with BD revealed poorer cognitive function than normal controls but no faster cognitive decline over 2 years [54]. This might partly be due to the relatively short interval between the two assessments. We suppose that our findings of larger cognitive deficits in older patients represent an age-related, accelerated cognitive decline, which may be a consequence of an ongoing neurodegenerative process in a subgroup of BD patients [21]. The present cross-sectional study does not indicate whether the described deficits are limited to the major depression or persist at the same magnitude into remission. It cannot be excluded that our finding of an age-related increased cognitive deficit in BD patients with severe depression is state dependent, and not indicative of a general age-related reduced cognitive capacity in euthymic BD. Our findings could indicate that older BD patients are more vulnerable to the development of cognitive deficits when depressed, possibly as a result of a reduced capacity to maintain cognitive functioning in a state of depression.

The present sample of highly selected, treatment-resistant patients might not be representative of less severely affected BD patients. Patients in this study may belong to a subgroup with progressive cognitive decline.

The results of this study indicate a strong association between age and cognitive deficits, but no association with any of the illness characteristics. The stronger influence of age compared to illness characteristics on cognitive functioning might reflect patients’ difficulties in recalling their illness history. Another possible explanation for our finding of a stronger influence of age than any of the illness severity measures could be that BD per se—independently from clinical factors—results in a greater vulnerability to age-related pathological processes.

The present study was subject to some limitations. Since it had a cross-sectional design, the relationship between age and accelerated cognitive decline should be confirmed in a longitudinally designed study. Although there are possible confounding effects from previous and current use of psychotropic medications, the medication at admittance did not differ significantly between the two groups. The sample size in our study limits both the use of regression models and the probability to detect possible significant differences in MCCB-scores between the bipolar subtypes (type II error); therefore, the negative findings of this study should be confirmed by larger studies. Data on the course of illness, such as the number of previous episodes, were collected retrospectively. Even if the data were supplied by information from significant others and hospital records there might have been a recollection bias. A selection bias might have been present due to recruitment through the Norwegian ECT study. All patients had to accept possible randomization to ECT and may therefore not be representative of all patients with treatment-resistant BD depression. In the present cross-sectional study the influence of the mood state on cognitive functions in BD was not examined. Further studies examining cognitive performance during illness episodes and in remission are warranted to address this issue.

The present study also has particular strengths: all participating study centers included patients from their defined catchment areas, all Norwegian acute psychiatric services are public and available to everyone, and all of the patients in the catchment areas were admitted to the local study center.

Cognitive impairment in BD depression must be addressed in clinical practice. Patients with cognitive impairments as described in the present study will probably experience difficulties in situations that demand rapid processing of information such as following complex instructions, sustaining attention, and remembering new information. This may result in problems dealing with practical tasks in daily life, work, maintaining social relationships, and treatment adherence. Neuropsychological functioning should be assessed routinely in BD depression in order to identify particular strengths and difficulties for the individual patient and adapt therapeutic strategies.

## Conclusions

A high proportion of patients with therapy-resistant BD I or II depression exhibited global neurocognitive impairments with clinically significant severity. The cognitive impairments were more common in BD I compared to BD II patients, particularly processing speed. These findings suggest that clinicians should be aware of the severe neurocognitive dysfunction in treatment-resistant bipolar depression, particularly in BD I.

## Competing interests

OA has received speaker’s honorarium from Lundbeck, BMS, GSK, Astra Zeneca and Janssen. UM has received speaker’s honorarium for lecturing about mood disorders from Astra Zeneca, GSK, Lundbeck, Norwegian Association for Hospitals, Norwegian Psychiatric Association, and received payment by the Norwegian Directorate of Health for serving as expert providing national guidelines for treatment of bipolar disorders and non-bipolar depression. GM has received speaker’s honorarium and travel grants from Astra Zeneca and Eli Lilly. All other authors report they have no conflicts of interest.

## Authors’ contributions

UK, HS, OA, ÅH, UM, KO, GM and AV conceived the research hypotheses and designed the study. KS contributed to the design of the study and the interpretation of the data. AV, GM, HS, UK and KO included the patients. GE contributed with statistical advices in the design of the study and the analysis of the data. UK wrote the manuscript. All authors contributed to, read and accepted the final manuscript.

## Pre-publication history

The pre-publication history for this paper can be accessed here:

http://www.biomedcentral.com/1471-244X/13/105/prepub
